# Editorial: Case reports in surgical oncology: 2022

**DOI:** 10.3389/fonc.2024.1300868

**Published:** 2024-02-13

**Authors:** Riccardo Bertolo

**Affiliations:** Urology Unit, Azienda Ospedaliera Universitaria Integrata Verona, AUOI Verona, University of Verona, Verona, Italy

**Keywords:** surgical oncology, urology, case report, surgery, malignant neoplasm

Dear readership, it is my pleasure to present you the Research Topic published on *Frontiers in Oncology* and *Frontiers in Surgery* entitled “*Case Reports in Surgical Oncology: 2022*”. While editing this Research Topic, unique and/or rare cases coming from different fields of surgical oncology were selected among the submissions received. As a uro-oncologist, I am very proud to present you a selection of the 18 case reports of interest for the urologic surgeons out of the 55 submissions finally accepted. It is impressive to observe the interest and the productivity of urology researchers witnessed by such numbers. This is particularly true given the wide spectrum of diseases embraced by surgical oncology. I tried to summarize you in a few sentences the key messages from each of the articles featured in my editorial. An “organ-by-organ” sequence was followed.

Five submissions were focused on oncologic surgical interventions performed to treat diseases of the genitalia or the sacral area. Hao et al. reported the case of a 69-year-old man presenting with a growing, painless mass protruding from the penis. The patient underwent resection of the penile mass, followed by extended resection in the second operation. The diagnosis of leiomyosarcoma was verified by pathological examination. The authors underlined that immunohistochemical examination is essential for rendering this rare diagnosis. Yan et al. reported a case of superficial angiomyxoma in a 42-year-old male patient. The patient was admitted to hospital with a perineal mass found more than 1 year previously. A pelvic contrast-enhanced computed tomography scan confirmed the 6.0 cm × 8.6 cm × 4.5 cm with still clear borders seen below the penile corpus cavernosum in the perineum. Perineal mass excision was performed, and pathology report diagnosed perineal superficial angiomyxoma. Chen et al. presented a rare case of aggressive angiomyxoma in the scrotum of a 70-year-old man, underlining the importance of considering aggressive angiomyxoma in the differential diagnosis of myxoid neoplasms growing painless in male genital areas. Jiang et al. reported about the fourth ever published nuclear protein of the testis (NUT) carcinoma of probable ovarian origin. NUT is a rare subset of poorly differentiated, highly aggressive malignancy defined by NUTM1 gene rearrangements. The diagnosis is confirmed by immunohistochemistry. With their publication, the authors underlined how it is essential to think about this rare disease whenever an undifferentiated malignant neoplasm arises from the abdominopelvic cavity. Chen et al. presented the case of a 49-year-old male patient with a history of frequent and urgent urination for 2 weeks. Radiologic studies revealed a large cystic mass in the lower abdomen. The patient underwent abdominal laparotomy, which revealed a large cystic mass arising from the distal ileum invading the sigmoid mesocolon and the bladder apex. Partial resection of the ileum along with the tumor and the adjacent bladder was performed. Macroscopic examination revealed that the cystic mass contained a large amount of foul-smelling pus and a tumor-bowel fistula. The final pathology revealed an abdominal stromal tumor. Postoperative recovery was uneventful, and adjuvant imatinib mesylate 400 mg was administered daily. The authors underlined that this is a rare presentation of the disease.

Seven submissions were focused on surgeries for renal cell carcinoma (RCC), of which two about partial nephrectomy, three about radical nephrectomy, and two about metastasectomy in metastatic RCC. Starting with partial nephrectomy, Yu et al. presented the case of a 71-year-old female patient diagnosed with a 20 x 16 cm RCC of the solitary functioning kidney. This was an imperative indication for nephron-sparing surgery. The patient initially presented with hematuria and acute urinary tract obstructive anuria caused by renal calculi. She underwent nephron-sparing surgery with success. It was a pT3 renal cancer. At 26-month follow-up renal function recovered to baseline level. No relapse was detected. Zou et al. reported the case of a 64-year-old man who was diagnosed with an extremely rare case of localized RCC in the left kidney complicated with situs viscerum inversus totalis and abdominal cocoon. The patient underwent robot-assisted partial nephrectomy. Surgery was uneventful. As regarding radical nephrectomy, Ning et al. reported the case of a female patient with low back pain diagnosed with a 7 cm left renal cystic mass. The mass was diagnosed to be a mucinous cystadenocarcinoma of the kidney after robot-assisted radical nephrectomy. This is a rare renal epithelial tumor originating from the urothelium of the pelvis. Zhang et al. reported the case of a 40-year-old woman who presented Castleman’s disease arising in the renal sinus which resembled a RCC. Castleman’s disease is a rare benign lymphoproliferative disease that frequently involves the mediastinal thorax and the neck lymph nodes. It rarely affects extra-thoracic presentations, with even fewer presentations in the renal sinus. In this case, the patient underwent radical nephrectomy. Histological examination revealed hyperplastic lymphoid follicles in the renal sinus and was finally diagnosed as Castleman’s disease. The authors underlined that due to the low incidence of Castleman’s disease at the level of the renal sinus, there is a strong likelihood of missing the diagnosis. Finally, Courcier et al. reported about a case of renal medullary carcinoma diagnosed in a 31-year-old male patient. It is a rare form of RCC with poor prognosis, known to be associated with sickle cell trait or disease, although the exact underlying mechanisms are still unclear. The diagnosis is made through immunochemical staining for SMARCB1 (INI1). The patient underwent upfront cisplatin-based cytotoxic chemotherapy before surgical removal of the right kidney and retroperitoneal lymph node dissection. Identical adjuvant chemotherapy was administered post-surgery. The disease relapses were detected in the retroperitoneal lymph nodes; these were managed with chemotherapy and surgical rechallenges until the patient died after 37 months of follow-up. The authors underlined that current management of renal medullary carcinoma relies on perioperative cytotoxic chemotherapy strategies, given that there are no known alternative therapies that have been shown to be superior to date. As aforementioned, the last two reports focused on metastatic RCC and specifically about surgical resection of unusual metastases of RCC. Shepherd et al. retrospectively analyzed 20 years of electronic records at their institution and reported about 5 patients with metastases of clear cell RCC to the thyroid. This is an uncommon location for RCC metastases, and the authors underlined about the importance to be aware they can occur. Yang et al. presented the case of a 84-year-old man who had small bowel intussusception and obstruction due to a solitary metachronous metastasis from RCC. The solitary metachronous small bowel metastases from RCC are rare. In contrast to idiopathic intussusception frequently occurring in children, adult intussusceptions are uncommon and usually indicate the presence of a malignant neoplasm. Surgical resection was performed with success. With their case, the authors underlined that life-long follow-up of RCC patients is critical due to the unpredictable behavior of the disease and the possibility of a long period of dormancy. Surgical resection is the mainstay treatment for such patients.

Four submissions were focused on surgeries for adrenal masses or at least of excision of unusual masses located in the adrenal lodge.

Feng et al. reported the case of a giant cystic pheochromocytoma in a 64-year-old woman discovered as a right abdominal mass during ultrasonography. This is the largest pheochromocytoma ever documented in China (20 cm in diameter). Atrophy of the right lobe of the liver was found at preoperative imaging and confirmed during surgical resection. The same group reported about laparoscopic resection of an adrenal myelolipoma, a commonly benign, asymptomatic, and hormonally non-secreting disease. They underlined that there is insufficient awareness of this adrenal incidentaloma among clinicians. Studies for establishing common guidelines in the management of adrenal myelolipoma are needed [Feng et al.]. Zou et al. reported the case of a 55-year-old patient incidentally diagnosed with a soft-tissue mass located in the left adrenal region. No specific abnormalities in biochemical indexes were found but the patient had a 10-year history of hypertension. The patient had undergone open splenectomy 20 years before for splenic rupture caused by traffic-accident trauma. Because of the uncertain nature of the mass, surgical treatment was recommended. During the surgery, the adrenal origin was excluded. At final pathology, the splenic corpuscle and splenic medullary structure were seen, so that an accessory spleen was diagnosed. With their report, the authors underlined that, although the diagnosis of adrenal tumours mainly depends on imaging, misdiagnosis can occur for some adrenal space-occupying lesions without specific signs/symptoms or abnormal biochemical indexes. Finally, Li et al. reported about a rare case of retroperitoneal bronchogenic cyst in a 57-year-old woman who was admitted with no clinical symptoms and found to have masses in the adrenal gland area during a routine physical examination. Abdominal computed tomography revealed a cystic lesion found in the left suprarenal region. The patient underwent a laparoscopic exploration. The histopathological finding confirmed the diagnosis of a retroperitoneal bronchogenic cyst. The authors underlined how such disease should be considered as one of the differential diagnoses among the retroperitoneal neoplasms.

The last two reports featured covered surgical interventions for other retroperitoneal neoplasms. Namely, Wang et al. reported about the rare association between acromegalic facial features induced by a retroperitoneal hemangiopericytoma and non-islet cell tumor hypoglycemia, a rare cause of hypoglycemia caused by the overproduction of high molecular weight insulin-like growth factor (big-IGF2), which activates the insulin receptor and subsequently caused hypoglycemia. The patient underwent complete surgical resection of the diagnosed mass. Surgical pathology demonstrated a hemangiopericytoma and strong positive for IGF-2. Lastly, Su et al. reported the case of a giant retroperitoneal cystic lymphangioma. Cystic lymphangioma is a rare benign tumor of the lymphatic system, which is most observed in the neck, head, and armpit. Less than 5% of lymphangiomas occur in the abdominal cavity and even less in the retroperitoneum. The patient underwent exploratory laparotomy, and the tumor was completely removed.

This is just a taste of what you can read in this Research Topic of unique case reports in urologic oncology. I hope I have intrigued you. Please feel free to enjoy each of the summarized submission in its full text format, which is open-access. You will definitely find interesting figures, including imagings, frames from intraoperative views, and photos of anatomo-pathology specimens when going through the articles. I sincerely feel that you will appreciate the very special clinical case scenarios in the present Research Topic.

## Author contributions

RB: Writing – original draft, Writing – review & editing.

